# Highly Linear Polyethylenes Achieved Using Thermo-Stable and Efficient Cobalt Precatalysts Bearing Carbocyclic-Fused *NNN*-Pincer Ligand

**DOI:** 10.3390/molecules24061176

**Published:** 2019-03-25

**Authors:** Jingjing Guo, Zheng Wang, Wenjuan Zhang, Ivan I. Oleynik, Arumugam Vignesh, Irina V. Oleynik, Xinquan Hu, Yang Sun, Wen-Hua Sun

**Affiliations:** 1 Beijing Key Laboratory of Clothing Materials R&D and Assessment, Beijing Engineering Research Center of Textile Nanofiber, School of Materials Science and Engineering, Beijing institute of Fashion Technology, Beijing 100029, China; jingguo1995@163.com; 2 Key Laboratory of Engineering Plastics and Beijing National Laboratory for Molecular Science, Institute of Chemistry, Chinese Academy of Sciences, Beijing 100190, China; wangzheng@iccas.ac.cn (Z.W.); avignesh@iccas.ac.cn (A.V.); sy0471103@iccas.ac.cn (Y.S.); 3 N.N. Vorozhtsov Novosibirsk Institute of Organic Chemistry, Pr. Lavrentjeva 9, Novosibirsk 630090, Russia; olk@nioch.nsc.ru; 4 College of Chemical Engineering, Zhejiang University of Technology, Hangzhou 310014, China

**Keywords:** highly linear polyethylene, vinyl-end polyethylene, cobalt precatalyst, thermo-stable and efficient catalysis, correlation between structure and activity

## Abstract

Six examples of 2-(1-arylimino)ethyl-9-arylimino-5,6,7,8-tetrahydrocycloheptapyridine-cobalt(II) chloride complexes, [2-(1-ArN)C_2_H_3_-9-ArN-5,6,7,8-C_5_H_8_C_5_H_3_N]CoCl_2_, (Ar = 2-(C_5_H_9_)-6-MeC_6_H_3_ **Co1**, 2-(C_6_H_11_)-6-MeC_6_H_3_ **Co2**, 2-(C_8_H_15_)-6-MeC_6_H_3_ **Co3**, 2-(C_5_H_9_)-4,6-Me_2_C_6_H_2_ **Co4**, 2-(C_6_H_11_)-4,6-Me_2_C_6_H_2_ **Co5**, and 2-(C_8_H_15_)-4,6-Me_2_C_6_H_2_ **Co6**), were synthesized by the direct reaction of the corresponding *ortho*-cycloalkyl substituted carbocyclic-fused bis(arylimino)pyridines (**L1**–**L6**) and cobalt(II) chloride in ethanol with good yields. All the synthesized ligands (**L1**–**L6**) and their corresponding cobalt complexes (**Co1**–**Co6**) were fully characterized by FT-IR, ^1^H/^13^C-NMR spectroscopy and elemental analysis. The crystal structure of **Co2** and **Co3** revealed that the ring puckering of both the *ortho*-cyclohexyl/cyclooctyl substituents and the one pyridine-fused seven-membered ring; a square-based pyramidal geometry is conferred around the metal center. On treatment with either methylaluminoxane (MAO) or modified methylaluminoxane (MMAO), all the six complexes showed high activities (up to 4.09 × 10^6^ g of PE mol^−1^ (Co) h^−1^) toward ethylene polymerization at temperatures between 20 °C and 70 °C with the catalytic activities correlating with the type of *ortho*-cycloalkyl substituent: Cyclopentyl (**Co1** and **Co4**) > cyclohexyl (**Co2** and **Co5**) > cyclooctyl (**Co3** and **Co6**) for either R = H or Me and afforded strictly linear polyethylene (*T*_m_ > 130 °C). The narrow unimodal distributions of the resulting polymers are consistent with single-site active species for the precatalyst. Furthermore, compared to the previously reported cobalt analogues, the titled precatalysts exhibited good thermo-stability (up to 70 °C) and possessed longer lifetime along with a higher molecular weight of PE (*M*_w_: 9.2~25.3 kg mol^−1^).

## 1. Introduction

**Chart 1:**
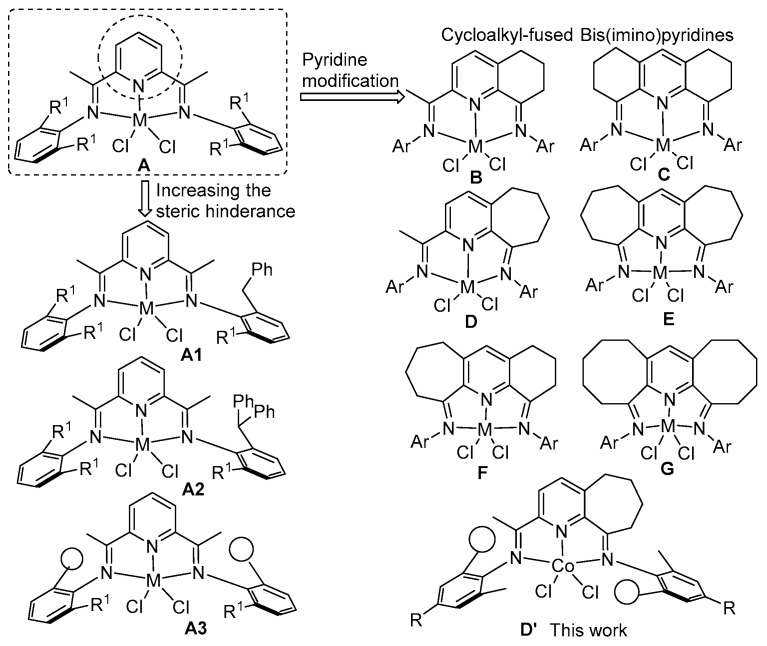
Bis(imino)pyridine cobalt complexes and cycloalkyl ring fused derivatives.

Over the past two decades ago, Brookhart [1,2] and Gibson [3,4,5] independently discovered the well-defined bis(imino)pyridine-cobalt and -iron complexes (**A**) as highly active precatalysts for ethylene oligo-/polymerization. In the intervening years, large numbers of different skeletal modifications, such as changes to the *N*-aryl groups and the backbone of pyridine, have been made to the bis(imino)pyridine scaffolds (**A**, Chart 1) [6,7,8,9,10,11,12,13,14,15], culminating in improvements to both catalytic activity and thermal stability [6,16,17,18,19,20,21,22,23,24,25,26,27,28,29,30,31,32,33]. The related progress was collected in several reviews [6,9,10,11,14,15].

To enhance the thermal stability, one efficient method was introduced to the bulky substituent on the *ortho*-position of *N*-aryl group [6,7,8,9,10,11,12,13,14,15] (Chart 1). Wu’s group have reported that bis(imino)pyridine iron complexes (**A1**) bearing a bulky 2-methyl-6-*sec*-phenethyl group, can produce linear high molecular weight polyethylene with higher activity at elevated temperature [31]. In the recent five years or so, our group have developed series of dibenzhydryl substituted bis(imino)pyridyl iron and cobalt complexes (**A2**) that displayed good catalytic activity at high temperature and explored a very good thermal stability [34]. There were also reports about incorporating the cycloalkyl substituents in the 2,6-positions of the *N*-aryl groups of the bis(imino)pyridine (**A3**) and it has been found to efficiently increase the temperature stability of the iron catalysts over their alkyl analogues [35]. Later Russia’s group documented the 2,6-bis(arylimino)pyridy liron(II) chloride complexes by the introduction of cycloaliphatic substituents into the *ortho* position of the aryl groups substantially enlarges the temperature interval of efficient use of the related catalysts for ethylene polymerization reactions [36]. Consequently, they have prepared the multifunctional bis(imino)pyridine iron chloride complexes incorporating the cycloalkyl substituent that could display more efficient ethylene polymerization at elevated temperatures (60–90 °C) while retaining high molecular weights of the resulting PE, as distinct from the analogous monofunctional systems (**A3**) [37].

On the other hand, the modification of bis(imino)pyridine backbone to enhance the catalytic properties at a higher temperature. Our group has been interested in introducing cycloalkyl unit to bis(imino)pyridine ligand sets by adjusting the flexibility of the exterior imine donors to modify the donor properties of the *NNN*-pincer ligand set and in-turn the performance of the catalyst [6]. With regards to cobalt precatalysts (Chart 1), one [38,39,40,41,42] or two [43,44,45,46,47,48] cycloalkyl unit fused derivatives having ring sizes between five and eight are accessible (see for example **B** [39], **C** [40], **D** [41], **E** [43,44], **F** [45], and **G** [47,48], Chart 1). In terms of catalytic performance and the polymeric products properties, we have observed significant differences between cobalt catalysts (**B**–**G**). For example, five-membered ring fused bis(imino)pyridine cobalt precatalyst [38], showed lower activities (2.89 × 10^4^ g of PE mol^−1^(Co) h^−1^) than their analogues of **A** and **B**–**G** [6]; while using **B** that containing slightly larger six-membered ring (Chart 1) [39], results in much higher activities (up to 1.08 × 10^7^ g of PE mol^−1^ (Co) h^−1^) and better thermal stability than **A** and **C** [4,6,39,40], and produces polyethylene wax with narrower molecular weight distribution. Expanding the ring size to seven (**D** and **E**, Chart 1) [41,43,44], the more flexible structure, leads to increased molecular weight (*M*_w_ up to 54.0 kg mol^−1^) which is most apparent with doubly-fused **E** containing *ortho*-cycloalkyl substituent, but **E** display slightly lower activity their counterparts **D**. Beyond this, it was found that cyclohexyl fused pyridine cobalt complexes (**B** and **C**) produced the polyethylene with very low molecular weight (0.82 kg mol^−1^) and linear saturated structure. The seven-membered ring fused pyridine(imino)cobalt precatalyst (**D** and **E**) produced the higher molecular weight polyethylene (3.2 kg mol^−1^) that contained the vinyl group, which has a significant potential application in the polywax in the package field [6]. The other interesting is the one cycloalkyl fused pyridine (imino) cobalt (**B** and **D**) showed better thermal stability (optimum temperature: 50~60 °C) and higher activity (8.65–10.09 × 10^6^ g of PE mol^−1^(Co) h^−1^) than that by double cyclcoalkyl fused bis(imino)pyridine cobalt complexes (**C**, **E**, **F**, and **G**) (activity: 2.89–5.04 × 10^6^ g of PE mol^−1^(Co) h^−1^ and optimum temperature at 30–40 °C). Very recently, we have reported a series of **E** derivatives that incorporate the cycloalkyl substitution on the *ortho* position of *N*-aryl group, which exhibited good activity (2.0 × 10^6^ g of PE mol^−1^(Co) h^−1^) with the optimum temperature of 30 °C [43]. Considering the potential positive effect by the cycloalkyl substituent on the *ortho* position of *N*-aryl group and the backbone of monocycloheptyl fused pyridine(imino) on the thermal stability and polymerization activity toward ethylene polymerization, therefore, in this work we focused on the mono cycloheptyl-fused bis(imino)pyridine cobalt complexes that incorporated the different ring size of cycloalkyl on the *ortho* position of *N*-aryl group and their catalytic behavior toward ethylene polymerization in detail.

## 2. Results

### 2.1. Synthesis and Characterization of Ligands and Cobalt Complexes **Co1**–**Co6**

**Scheme 1:**
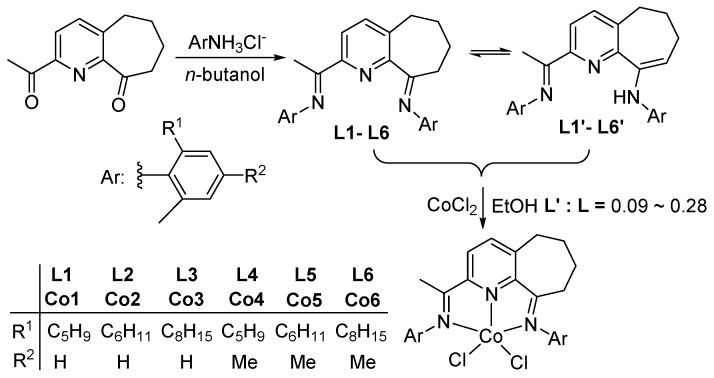
Synthetic route of ligands **L1**–**L6** and cobalt complexes Co1–Co6.

According to a literature procedure [49], the condensation reactions of 2-acetyl-5,6,7,8-tetrahydrocycloheptapyridin-9-one with 2-cycloalkylaniline hydrochloride derivatives were conducted in the refluxing *n*-butanol to form the desired *NNN*-pincer ligands (**L1**/**L1’**–**L6**/**L6’**, Chart 1) in moderate isolated yield (32%–39%, Scheme 1).

The ^1^H-NMR spectra of the ligands indicated two isomers for each because of the migration of double bond to the imino group linked to the cycloheptane, illustrating major compounds with the remaining imino group (**L**) instead of double bonds within cycloheptanic derivatives (**L′**, minor), which is similar to our previous work [49,50]. The isomers with N–H groups are confirmed with the observation range from 3372 to 3379 cm^−1^ in their IR spectra, while the isomers containing the C=N groups were observed characteristic band around 1644 cm^−1^. Then using the literature procedure [40,49], the corresponding *NNN*-pincer ligands (**L1**/**L1’**–**L6**/**L6**) reacted with cobalt(II) dichloride in ethanol to give the corresponding 2-(1-arylimino)ethyl-9-arylimino-5,6,7,8-tetrahydrocyclohepta -pyridine-cobalt(II) chloride complexes, [2-(1-ArN)C_2_H_3_-9-ArN-5,6,7,8-C_5_H_8_C_5_H_3_N]CoCl_2_, (Ar = 2-(C_5_H_9_)-6-MeC_6_H_3_ **Co1**, 2-(C_6_H_11_)-6-MeC_6_H_3_ **Co2**, 2-(C_8_H_15_)-6-MeC_6_H_3_ **Co3**, 2-(C_5_H_9_)-4,6-Me_2_C_6_H_2_ **Co4,** 2-(C_6_H_11_)-4,6-Me_2_C_6_H_2_ **Co5**, and 2-(C_8_H_15_)-4,6-Me_2_C_6_H_2_ **Co6**), in high yields (91–96%). All the ligands **L1**–**L6** have been characterized by FT-IR, ^1^H/^13^C-NMR spectroscopy and elemental analysis. The cobalt complexes **Co1**–**Co6** have been characterized by IR and elemental analysis and the structure of **Co2** and **Co3** was confirmed by single-crystal X-ray diffraction. Comparing the IR spectra of free ligands with their corresponding Co complexes, there is no absorption related to the N–H groups; moreover, the adsorption around 1644 cm^−1^ for ν_C=N_ in ligand compounds is shifted to lower wavenumber around 1609 cm^−1^ within the cobalt complexes indicating effective coordination of Co–N_sp2_ [40,41].

### 2.2. Single-Crystal X-ray Diffraction Study

**Figure 1:**
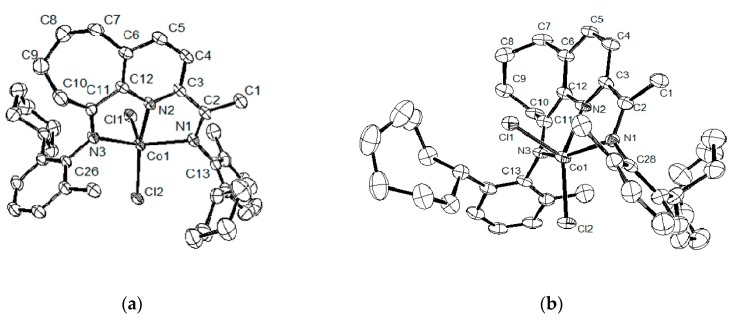
ORTEP drawing of **Co2** (**a**) and **Co3** (**b**) with thermal ellipsoids at the 30% probability level. Hydrogen atoms are omitted for clarity.

The single-crystals of **Co2** and **Co3** suitable for the X-ray determinations were obtained by slow diffusion of *n*-hexane into their solution in dichloromethane at room temperature. Their molecular structures are shown in Figure 1a,b, and selected bond lengths and angles in Table 1.

**Co2** and **Co3** have a similar structure and each cobalt was surrounded by two chlorides and three nitrogen atoms belonging to the corresponding *NNN*-pincer ligand. Both of them possessed distorted square-pyramidal geometry, in which three nitrogen atoms N1, N2, N3 and Cl1 formed the base plane and Cl2 occupying the apical position. The cobalt atom lies at a distance of 0.505 Å above the basal plane for **Co2** and 0.447 Å for **Co3** in a manner similar to that observed in related NNNCoCl_2_ counterparts [40,41,45,47]. In these two structures, the bond length of Co-N_py_ [2.063(4) Å (**Co2**), 2.043(1) Å (**Co3**)] is significantly shorter than that of Co-N_imine_ [2.203(4) and 2.183(3) Å (**Co2**), 2.193(4) and 2.175(4)Å (**Co3**)], suggesting the stronger donor ability by N-py. At the same time, they are close to that in **A** (2.051(3) Å) [4], **B** (2.040(3) Å) [39], **D** (2.036(4)–2.052(5) Å) [40,41], but are shorter than that of **E** (2.087(4) Å) [44]. By contrast, the bond length of Co–N_imine_ [2.175(4)–2.203(4) Å] are longer than that in **E** (2.128(3) and 2.176(3) Å) [51] and α,α′-bis(arylimino)-2,3:5,6-bis(hexamethylene)pyridine-cobalt(II) complexes (range: 2.1180(18) - 2.1633(18) Å) [47], similar to those **A** [4], **B** [39], and **D** [40,41] (range: 2.193–2.320 Å, Chart 1), highlighting the good chelation properties of **D** to the cobalt center. The *N*-aryl rings are almost perpendicular to the coordination plane with dihedral angles of (74.23° and 78.48°) for **Co2** and (83.84° and 71.39°) for **Co3**, respectively. Concurrently, the cyclo-substituents always located in *trans*-position. The torsion angles for N(1)–C(2)–C(3)–N(2) [2.09° (**Co2**), −7.08° (**Co3**)] and N(3)–C(11)–C(12)–N(2) [−10.57° (**Co2**), 13.35° (**Co3**)] highlight the deviation from co-planarity between the pyridine ring and the neighboring imine vectors, and the large distinct torsion angles are attributed to the unsymmetrical framework and the different sizes *ortho*-cycloalkyl substituent [44]. The interesting is the substituents on the *ortho* position of N-aryl group are always in *trans*-position.

### 2.3. Ethylene Polymerization

Based on previous findings for structurally related cobalt (II) chloride complexes (e.g., **B**–**E** in Chart 1) [39,40,41,42,43,44,45,46,47], the highest catalytic activity for ethylene polymerization tends to be achieved upon activation with either methylaluminoxane (MAO) or modified methylaluminoxane (MMAO) [6,7,8,9,10,11,12,13,14,15]. Therefore, these two co-catalysts were employed to investigate the ethylene polymerization in detail as follows.

#### 2.3.1. Ethylene Polymerization using **Co2**/MAO

With MAO as co-catalyst, **Co2** was employed as precatalyst to investigate the ethylene polymerization systematically at various parameters, such as different Al/Co molar ratios, reaction temperatures and run times and polymerization results are collected in Table 2.

**Figure 2:**
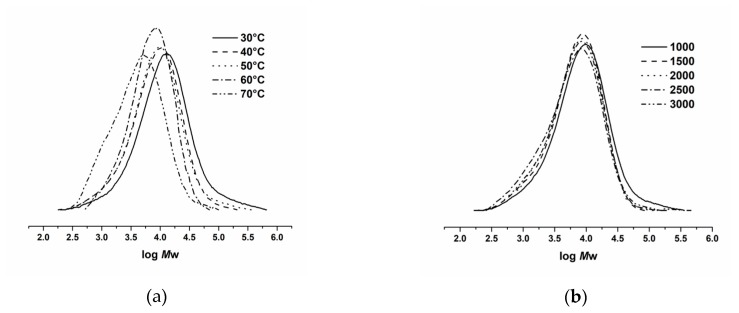
(**a**) GPC curves of polyethylene obtained by **Co2**/MAO at various temperature (runs 1–5, Table 2); (**b**) GPC curves of polyethylene obtained using **Co2**/MAO with different molar ratio of Al/Co (runs 3 and 6–11, Table 2).

First of all, with the ethylene pressure set at 10 atm and the Al/Co ratio fixed at 1000, ethylene polymerizations were conducted at a different temperature from 30 to 70 °C (runs 1–5, Table 2). The maximum activity of 2.89 × 10^6^ g of PE mol^−1^ (Co) h^−1^ was found at 50 °C affording a polymeric product; no trace of oligomers could be detected. Further, higher temperature led to the dramatically decreasing of the polymerization activity (Figure 2a). Meanwhile, the molecular weight (*M*_w_) of the polymer was found to decrease from 22.97 kg mol^−1^ to 6.56 kg mol^−1^ when increasing the temperature from 30 °C to 70 °C, which can be ascribed to either the more facile chain transfer or chain termination with respect to the chain propagation yielding lower molecular weight PE [4,40,41,45,51,52,53]. Moreover, their GPC curves showed the unimodal distribution and the resultant polyethylene possessed narrow polydispersity (PDI = 2.0~3.8), which is consistent with the characters of single-site active species [6,39,40,41,42,43,44,45].

Fixing the temperature at 50 °C, ethylene polymerizations by **Co2**/MAO were conducted at a different Al/Co molar ratio from 500 to 3500 (runs 3 and 6–11, Table 2). The highest activity was observed as 3.57 × 10^6^ g of PE mol^−1^(Co) h^−1^ with a molar ratio of 2500 (run 9, Table 2), a lower or higher ratio of co-catalyst led to lower activities. The molecular weight of the polymer (*M*_w_) gradually decreased from 14.2 kg mol^−1^ to 9.39 kg mol^−1^, and the molecular weight distribution kept very narrow (PDI = 2.4 − 2.7). This result can be ascribed to increased chain transfer to aluminum occurring as a consequence of the larger amounts of alkyl aluminum reagent employed [4,40,41,44,51,52]. To our surprise with the Al/Co molar ratios changing between 3000 and 3500, a dramatic increase of molecular weight was observed and up to 13.96 kg mol^−^^1^ with 3500 equivalents of MAO (Figure 2b). It would seem plausible that the formation of new active species has been influenced within this molar ratio window, a similar observation has been noted elsewhere [38,44]. On the other hand, with regards to the molecular weight distribution, the PDI of polymers are falling in the range from 2.4 to 3.0, slightly broader than their counterparts **D** containing symmetric *para*-R^1^ groups (PDI = 2.2) [41], this observation was similar to complex **E** incorporating α, α′-bis(arylimino)-2,3:5,6-bis(hexamethylene)pyridine containing cycloalkyl *ortho*-substituents [43,44].

To the end, the lifetime of the **Co2**/MAO catalyst was probed by conducting the polymerization over 5, 15, 30, 45 and 60 min (runs 9 and 12–15, Table 2). Polymerization activity gradually decreased from 8.12 × 10^6^ g of PE mol^−1^(Co) h^−1^ to 2.48 × 10^6^ g of PE mol^−1^ (Co) h^−1^ with prolonging the reaction time from, 5 to 60 min, which can be explained by the rapid formation of active species after the addition of MAO and gradual deactivation over extended reaction time [34,40,41,42,43,44,45]. It is indicated that a short time frame was required to generate all active species, and then the onset of partial deactivation of active species occurred over the course of the reaction [34]. Decreasing ethylene pressure to 5 atm, the polymerization activity (2.85 × 10^6^ g of PE mol^−1^ (Co) h^−1^) is much lower than that (3.57 × 10^6^ g of PE mol^−1^ (Co) h^−1^) at 10 atm (runs 9 and 16, Table 2), but the resultant PE polymer possessed much higher molecular weight (13.04 vs. 9.39 kg mol^−1^).

**Figure 3:**
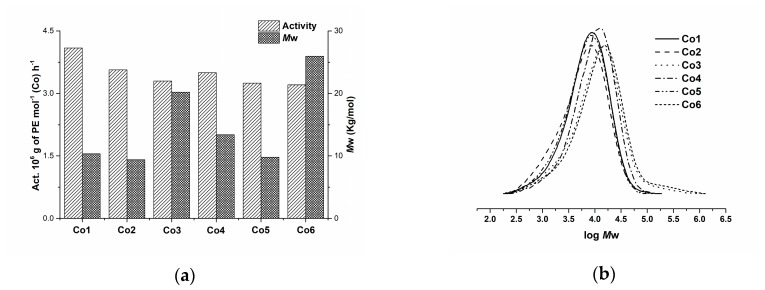
(**a**) Polymerization activity and *M*_w_ of PE by **Co1-Co6**/MAO; (**b**) GPC curves of PE obtained by **Co1-Co6**/MAO (runs 9 and 17–21, Table 2).

In order to explore the effect imparted by the cycloalkyl *ortho*-substitution pattern on performance and polymer properties, the other complexes were additionally screened using the optimized reaction conditions established independently for **Co2**/MAO and the results were collected in Table 2 (runs 17–21). On activation with MAO, all the cobalt complexes **Co1**–**Co6** displayed good activities in the range of 3.21–4.09 × 10^6^ g of PE mol^−1^ (Co) h^−1^, which fall in the order: **Co1** [R^1^ = cyclopentyl] > **Co2** [R^1^ = cyclohexyl] > **Co3** [R^1^ = cyclooctyl], **Co4** [R^1^=cyclopentyl] > **Co5** [R^1^ = cyclohexyl] > **Co6** [R^1^ = cyclooctyl] (Figure 3a). These findings indicate that the size of cycloalkyl group on the *ortho* position of N-aryl group affects the catalytic activity, in which less bulky cyclopentyl systems gave the higher activities (**Co1**, **Co4**) and the bulkiest group of cyclooctyl showed the lowest (**Co3**, **Co6**) activities. All the resultant PE by different cobalt complexes **Co1**–**Co6** possessed narrow distribution and PDI value ranged from 2.4 to 3.9, a slightly broader than that found in the **D**/MAO system. The GPC traces clearly showed unimodal distribution (Figure 3). But the polymer by **Co3** and **Co6** that bear the bulkiest *ortho*-substituent of cyclooctyl possessed the highest molecular weight among their analogues, the similar trends were also observed for **E**/MAO system [44]. The *para*-methyl substituted complexes **Co4**–**Co6** showed slightly lower activity than unsubstituted ones **Co1**–**Co3**, which was demonstrated by the activity order: **Co1** (R^2^ = H) > **Co4** (R^2^ = Me), **Co2** (R^2^ = H) > **Co5** (R^2^ = Me) and **Co3** (R^2^ = H) > **Co6** (R^2^ = Me). However, Figure 3 shows that molecular weights by **Co1**–**Co3** are lower than that by **Co4**–**Co6** respectively. These trends are similar to their analog complex of **B** [39].

**Chart 2:**
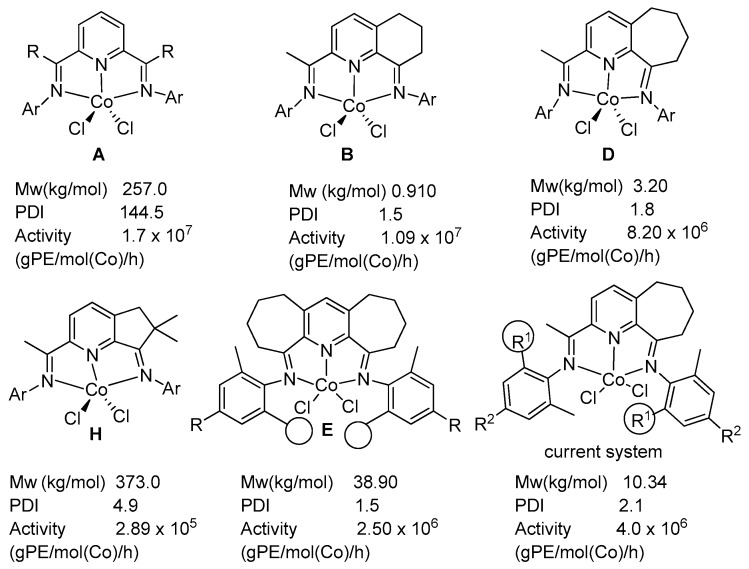
Comparison of the *M*_w_, polydispersity (PDI) and polymerization activity of previously reported cobalt precatalysts (A [4], B [39], D [40], E [44] and H [38]) with MAO as activator under related condition.

In comparison with previously reported cobalt precatalysts, such as **A**, **B** and **D** (Chart 2) [4,39,40,41,44], the current systems, **Co1**–**Co6**, under comparable polymerization conditions (namely MAO as co-catalyst, 10 atm C_2_H_4_, 30 min) exhibited relatively lower catalytic activity (3.21–4.09 × 10^6^ g of PE mol^−1^ (Co) h^−1^), but are higher than that by precatalyst **E** (Chart 2). On the other hand, the resulting polymers by **Co1**–**Co5**/MAO possessed much higher molecular weight than that found in **D**/MAO. These findings heights cycloalkyl *ortho*-substituents in the N-aryl group favored the high molecular weight polymer formation.

#### 2.3.2. Ethylene Polymerization Using **Co2**/MMAO

Under the optimized molar ratio for **Co2**/MAO (Al/Co = 2500), ethylene polymerization by **Co2**/MMAO were conducted at various temperatures (from 30 °C to 60 °C) at 10 atm ethylene pressure (runs 1–4, Table 2). The results showed that the highest activity of 2.95 × 10^6^ g of PE mol^−1^(Co) h^−1^ was also observed at 50 °C (run 3, Table 3), similar to the results by **Co2**/MAO (2.89 × 10^6^ g of PE mol^−1^ (Co) h^−1^). Further, higher temperature led to the decrease of activity, indicating the partial decomposition of the active species [4,40,41,45,51,52,53]. The molecular weight of PE decreased from 12.98 kg mol^−1^ to 6.41kg mol^−1^ with the increasing the temperature from 30 °C to 60 °C, which can be ascribed to either increased chain transfer to aluminum or chain termination by β-H elimination at the higher temperature [47,48,49,50,51,52,53,54]. However, all the resultant PE possessed quite narrow distribution (PDI = 2.5) and GPC traces showed unimodal distribution, which is a typical character of a single site catalyst system. A similar observation was also reported for their counterparts **B**, **D** and **E** (Chart 2).

With the temperature fixed at 50 °C, the influence of the Al/Co molar ratio was investigated in the range of 1500 to 3000 (runs 3, 5–7, Table 3). A topmost of activity 2.95 × 10^6^ g of PE mol^−1^(Co)h^−1^ was achieved at the molar ratio of 2500, which is similar to the results with MAO. As with MMAO, the resultant polyethylenes possessed high molecular weights (8.19–10.22 kg mol^−1^) and narrow polydispersities (*M*_w_/*M*_n_ ≈ 2.5), which is consistent with single-site characteristics for the active species [40,41,42,43,44,45].

In order to investigate the lifetime of the active species, the polymerization tests were conducted over different reaction time from 5, 15, 30 and 60 min (runs 3 and 8–10, Table 3). The results showed that polymerization activity decreased from 7.04 to 2.07 × 10^6^ g of PE mol^−1^ (Co) h^−1^ when increasing the time from 5 to 60 min. As with MMAO system, the molecular weights of the resultant polymer are falling in the range from 8.19 to 10.49 kg mol^−1^, and displaying narrow molecular weight distribution (*M*_w_/*M*_n_ = 2.4–3.0). With the ethylene pressure reduced to 5 atm, the activity (1.90 × 10^6^ g of PE mol^−1^ (Co) h^−1^) was much lower than that at 10 atm (run 8, Table 3).

**Figure 4:**
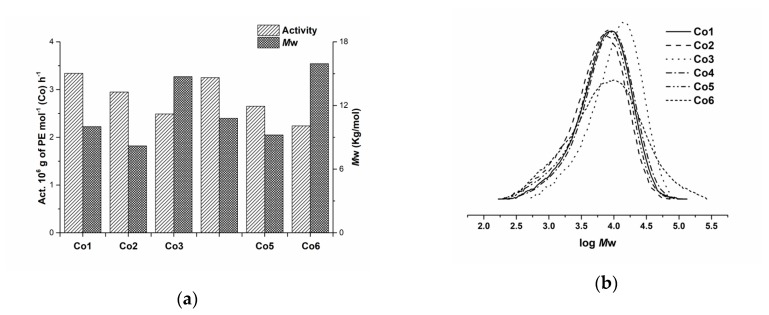
(**a**) Polymerization activity and *M*_w_ of PE by **Co1-Co6**/MMAO; (**b**) GPC curves of PE obtained by **Co1**–**Co6**/MMAO (runs 3 and 12–16, Table 3).

With MMAO as co-catalyst, all the cobalt complexes **Co1**–**Co6** were evaluated for their ethylene polymerization and they also displayed good activity (2.24–3.34 × 10^6^ g of PE mol^−1^ (Co) h^−1^). The similar trends in activity and molecular weight as MAO are observed, in which the cyclopentyl substituted **Co1** and **Co4** displayed the highest activity and cyclooctyl substituted cobalt complex (**Co3** and **Co6**) gave the lowest activity, but produced the highest molecular weight polyethylene (runs 3, 12–16, Table 3). In comparison with **D**/MMAO, the *ortho*-cycloalkyl systems in general exhibited more than twice higher molecular weight, although the catalytic activity of **D**/MMAO is nearly twice as much as that seen for **Co1** and **Co4**, and almost three times that for **Co2**–**Co3** and **Co5**–**Co6**. In addition, the molecular weights obtained using **Co1**–**Co6** (range: 8.19–15.94 kg mol^−1^) are all significantly higher than that seen for **D**/MMAO system (2.5 kg mol^−1^) [40], which also illustrated the steric effect of the *ortho* cycloalkyl substituents suppressed the chain transfer and led to the high molecular weight of the polymer. As shown by their GPC curves in Figure 4, the MWD of polymeric products are falling in the range 2.4 to 4.4.

### 2.4. Characterization of Polyethylene

**Figure 5:**
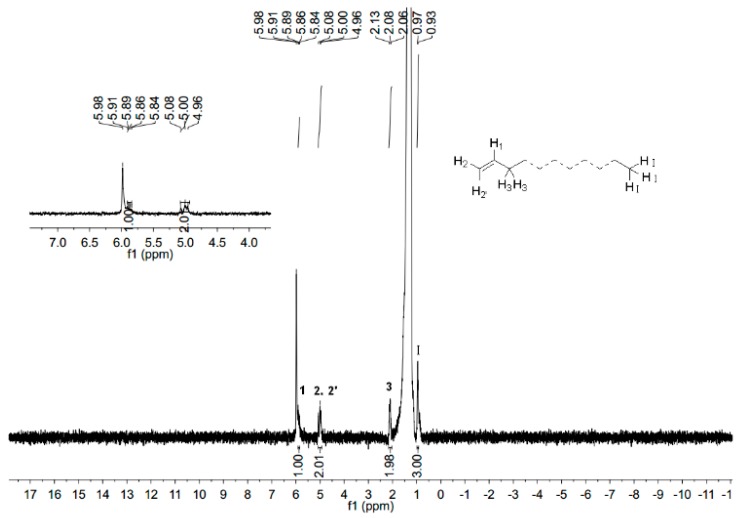
^1^H-NMR spectrum of the polyethylene obtained using **Co2**/MAO at 50 °C (run 9, Table 2); recorded in 1,1,2,2-tetrachloroethane-*d*_2_ at 120 °C.

**Figure 6:**
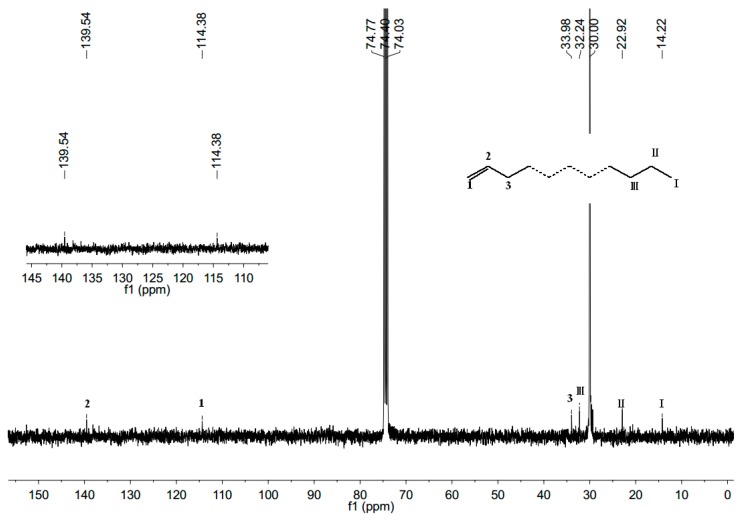
^13^C-NMR spectrum of the polyethylene obtained using **Co2**/MAO at 50 °C (run 9, Table 2); recorded in 1,1,2,2-tetrachloroethane-*d*_2_ at 120 °C.

Regardless of MMAO or MAO activation, narrow unimodal distributions of the polymers are observed (*M*_w_/*M*_n_ =2.2–4.5) consistent with single-site active species. All the *T*_m_ values of the polymer ranging from 129 °C to 133 °C [6,40,41,42,43,44,45]. To provide further support for the linearity of the polymers, representative PE samples using **Co1**/MAO at 50 °C (run 9, Table 2) were characterized by ^1^H/^13^C-NMR spectroscopy at high temperature (recorded in 1,1,2,2-tetrachloroethane-*d*_2_ at 120 °C, Figure 5 and Figure 6). The ^1^H-NMR spectra clearly showed the weak downfield multiplets at δ 5.90 and δ 5.00, the typical vinylic signal (Figure 5). While the integration ratio for H_1_:H_2_/H_2_’:H_I_ is close to 1:2:3, indicating the only the unsaturated chain structure in the PE. The shift 114.38 and 139.54 in ^13^C-NMR spectra was ascribed to the unsaturated chain ends (–CH=CH_2_) [16,40,41,43,45], which agreed well with ^1^H-NMR spectra. In addition, the lower intensity peaks at 32.24, 22.92 and 14.22 corresponding to an *n*-propyl end-group further supported the linear structure of the polyethylene [16,18,45]. These findings are further evidenced by their high melting temperature (*T*_m_ = 130.0 °C, run 9, Table 2).

**Figure 7:**
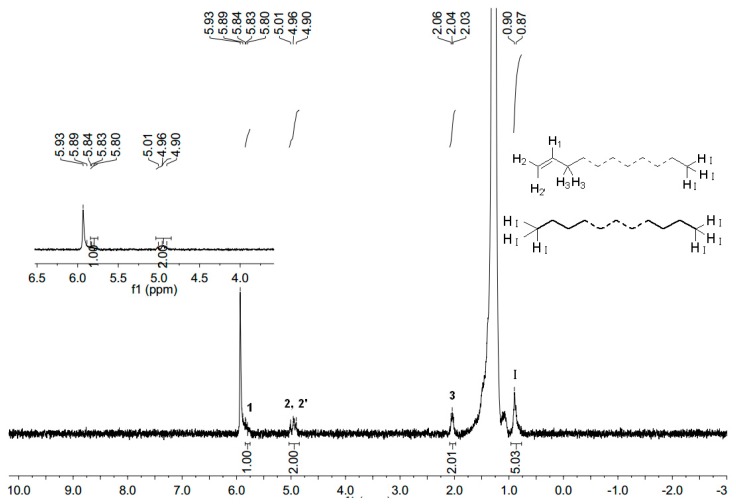
^1^H-NMR spectrum of the polyethylene obtained using **Co2**/MMAO at 50 °C (run 3, Table 3); recorded in 1,1,2,2-tetrachloroethane-*d*_2_ at 120 °C.

**Figure 8:**
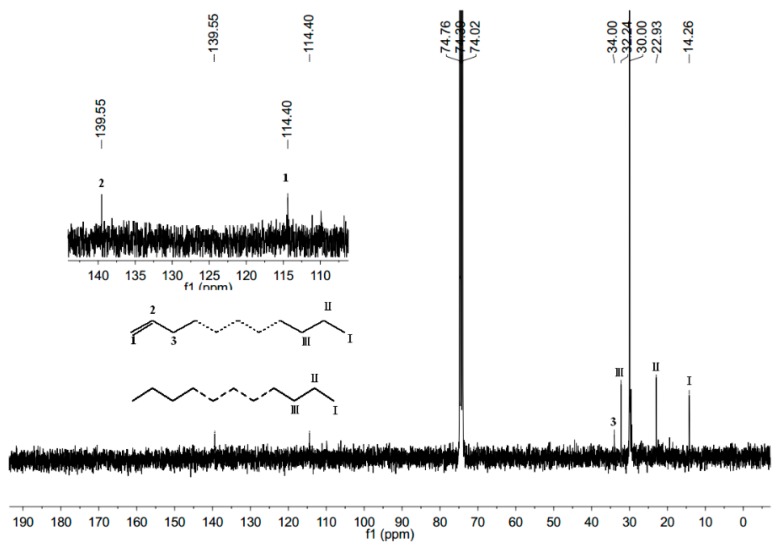
^13^C-NMR spectrum of the polyethylene obtained using **Co2**/MMAO at 50 °C (run 3, Table 3); recorded in 1,1,2,2-tetrachloroethane-*d*_2_ at 120 °C.

The resultant PE by **Co2**/MMAO were also characterized by ^1^H/^13^C-NMR spectra, and shown in Figure 7 and Figure 8. The ^1^H-NMR spectrum also showed the signal of a vinyl group (δ 5.90 and δ 5.00), which was also confirmed by the signal in ^13^C-NMR (δ 139.55, 114.40). However, the integration ratio for H_1_:H_2_/H_2_’:H_I_ is close to 1:2:5 (Figure 7), dissimilar to that seen with MAO at the same temperature, which suggested both the saturated and unsaturated chain structure in polyethylene. Moreover, the signal of the lower intensity peaks at 32.24, 22.93 and 14.26 in ^13^C-NMR corresponds to *n*-propyl end-group, indicating the linear structure of PE. These observations would suggest that, chain termination via β-H elimination in **Co2**/MMAO system is no longer the sole chain transfer pathway operative, with chain transfer to aluminum now competitive [16,18,44].

## 3. Materials and Methods

### 3.1. General Considerations

All the synthetic procedures for air/moisture-sensitive compounds were performed under a nitrogen atmosphere with standard Schlenk techniques. Toluene as a solvent for polymerization was refluxed over sodium (a small amount of benzophenone) and distilled under nitrogen prior to use. Methylaluminoxane (MAO, 1.46 M solution in toluene) and modified methylaluminoxane (MMAO, 1.93 M in *n*-heptane) were bought from Akzo Nobel Corp (Nanjing, China). High-purity ethylene was bought from Beijing Yanshan Petrochemical company (Beijing, China) and used as received. NMR spectra were recorded using a Bruker DMX 400 MHz instrument (Beijing, China) at ambient temperature using TMS as an internal standard. FT-IR spectra were recorded using a Perkin-Elmer System 2000 FT-IR spectrometer (Shanghai, China). Elemental analysis was carried out using a Flash EA1112 microanalyzer (Beijing, China). Molecular weights and molecular weight distribution of polyethylenes were determined using a PL-GPC220 GPC/SEC High Temperature System (Beijing, China). Data collection and handling were carried out using Cirrus GPC Software (Beijing, China) and Multi Detector Software (Beijing, China). The calibrants for constructing conventional calibration is Polystyrene Calibration KitS-M-10 from PL Company (Beijing, China). The true average molecular weights of PE are transferred by inputting the *M–H* constants of PE. K of 0.727 and α of 40.6 are provided by PL Company (Beijing, China). Samples were dissolved at a concentration of 1.0 to 2.5 mg mL^−1^, depending on the molecular weights. DSC trace and melting points of polyethylene were obtained from the second scanning run on DSCQ2000 at a heating rate of 10 °C min^−1^ from −20 °C to 200 °C. ^1^H/^13^C-NMR spectra of the polyethylene were recorded using a Bruker DMX 300 MHz instrument (Beijing, China) at 120 °C in deuterated 1,1,2,2-tetrachloroethane with TMS as an internal standard. The compound of 2-acetyl-5,6,7,8-tetrahydrocycloheptapyridin-9-one was synthesized according to the literature [49] and the 2-cycloalkylaniline hydrochlorides were prepared using literature methods [34].

### 3.2. Synthesis of 2-(1-ArN) C_2_H_3_-9-ArN-5,6,7,8-C_5_H_8_C_5_H_3_N (**L1**–**L6**)

Ar = 2-(C_5_H_9_)-6-MeC_6_H_3_ (**L1**/**L1′**). 2-cyclopentyl-6-methylphenylamine hydrochloride (0.46 g, 2.2 mmol) was added to a solution of (0.20 g, 1.0 mmol,) 2-acetyl-5,6,7,8-tetrahydrocycloheptapyridin-9-one with *p*-toluenesulfonic acid (20.0 mg) as catalyst in 30 mL of *n*-butanol. The mixture was stirred at reflux temperature for 6h. The solvent was then evaporated at reduced pressure, and the residue was purified by alumina column chromatography with petroleum ether/dichloromethane/triethylamine [100/1/1, *v*/*v*/*v*] to afford **L1** (0.19 g, 38.1%) as a yellow oil with molar ratio of **L1**/**L1′** = 1/0.12 (detected by ^1^H-NMR). ^1^H-NMR (CDCl_3_, 400 MHz, TMS): δ 8.39 (t, *J* = 7.1 Hz, 1H, **L1**–*H*_Py_), 8.25 (d, *J* = 8.2 Hz, 1H, **L1′**–*H*_Py_), 7.68 (d, *J* = 7.8 Hz, 1H, **L1′**–*H*_Py_), 7.62 (d, *J* = 8.0 Hz, 1H, **L1**–*H*_Py_), H), 7.18 (t, *J* = 8.5 Hz, 2H, 2 × **L1**–*H*_Ar_), 7.11 (t, *J* = 5.8 Hz, 2H, 2 × **L1′**–*H*_Ar_), 7.04 (t, *J* = 6.8 Hz, 2H, 2 × **L1**–*H*_Ar_ and 2 × **L1′**–*H*_Ar_), 6.99 (d, *J* = 7.3 Hz, 2H, 2 × **L1**–*H*_Ar_), 6.85 (d, *J* = 7.0 Hz, 2H, 2 × **L1′**–*H*_Ar_), 6.46 (s, 1H, **L1′**–*H*_NH_), 4.66 (t, *J* = 6.9 Hz, 1H, **L1′**–*H*_CH=_), 3.03-2.91 (m, 2H, **L1**–*H*_CH2_), 2.90–2.81 (m, 2H, **L1**–*H*_CH2_), 2.77 (t, *J* = 6.3 Hz, 2H, **L1′**–*H*_CH2_), 2.37 (s, 3H, **L1′**–*H*_CH3_), 2.34 (t, *J* = 6.1 Hz, 2H, **L1**–*H*_CH2_), 2.26 (s, 2H, **L1**–*H*_CH2_), 2.21 (s, 3H, **L1**–*H*_CH3_), 2.01 (d, *J* = 6.2 Hz, 6H, 2 × **L1**–*H*_PhCH3_), 1.95–1.88 (m, 2H, 2 × **L1**–*H*_CH2_), 1.82 (s, 4H, **L1**–*H*_CH2_), 1.72 (t, *J* = 5.7 Hz, 4H, **L1**–*H*_CH2_), 1.60 (d, *J* = 5.3 Hz, 4H, **L1**–*H*_CH2_), 1.54 (s, 4H, **L1**–*H*_CH2_). ^13^C-NMR (CDCl_3_, 100 MHz, TMS): δ 173.7, 167.5, 156.6, 155.0, 148.1, 137.4, 135.9, 134.4, 134.0, 128.1, 127.9, 125.5, 125.3 125.1, 124.1, 124.0,123.9, 123.5, 123.3, 121.6, 40.6, 40.5, 34.2, 34.1, 33.9, 33.7, 32.4, 31.9, 27.2, 26.3, 26.0, 25.9, 25.8, 24.1, 22.9, 18.5, 17.0, 16.9. FTIR (KBr, cm^−1^): 3372 (ν_N–H_, w), 2946 (s), 2864 (m), 1642(ν_C=N_, m), 1564 (w), 1455 (s), 1359 (m), 1302 (w), 1261 (m), 1192 (m), 1164 (w), 1118 (w), 1088 (m), 1032 (w), 848 (w), 805 (w), 746 (s). Anal. Calcd for C_36_H_43_N_3_: C, 83.51, H, 8.37, N, 8.12; Found: C, 83.18, H, 8.61, N, 8.43%.

Ar = 2-(C_6_H_11_)-6-MeC_6_H_3_ (**L2**/**L2′**). Using a similar procedure and molar ratios to that described for **L1**/**L1′** but with 2-cyclohexyl-6-methylphenylamine hydrochloride as the amine, **L2**/**L2′** was obtained as a yellow oil (0.22 g, 39.7%) with molar ratio of **L2**/**L2′** = 1/0.11 (detected by ^1^H-NMR).^1^H-NMR (CDCl_3_, 400 MHz, TMS): δ 8.38 (t, *J* = 8.5 Hz, 1H, **L2**–*H*_Py_), 8.24 (d, *J* = 7.8 Hz, 1H, **L2′**–*H*_Py_), 7.68 (d, *J* = 7.7 Hz, 1H, **L2′**–*H*_Py_), 7.63 (d, *J* = 7.6 Hz, 1H, **L2**–*H*_Py_), H), 7.14 (t, *J* = 8.1 Hz, 2H, 2 × **L2**–*H*_Ar_ and 2 × **L2′**–*H*_Ar_), 7.05 (m, 2H, 2 × **L2**–*H*_Ar_ and 2 × **L2′**–*H*_Ar_), 6.99 (d, *J* = 7.3 Hz, 2H, 2 × **L2**–*H*_Ar_), 6.94 (s, 2H, 2 × **L2′**–*H*_Ar_), 6.41 (s, 1H, **L2′**–*H*_NH–_), 4.66 (t, *J* = 6.9 Hz, 1H, **L2′**–*H*_CH=_), 2.96 (t, *J* = 6.0 Hz, 2H, **L2**–*H*_CH2_), 2.79–2.71 (m, 2H, **L2**–*H*_CH2_), 2.64 (s, 2H, **L2′**–*H*_CH2_), 2.38 (s, 3H, **L2′**–*H*_CH3_), 2.34 (t, *J* = 6.3 Hz, 2H, **L2**–*H*_CH2_), 2.28 (s, 2H, **L2**–*H*_CH2_), 2.21 (s, 3H, **L2**–*H*_CH3_), 2.01 (d, *J* = 5.0 Hz, 6H, 2 × **L2**–*H*_CH3-Ph_), 1.97 (s, 2H, 2 × **L2**–*H*_CH2_), 1.91–1.82 (m, 4H, **L2**–*H*_CH2_), 1.78–1.67 (m, 8H, 2 × **L2**–*H*_CH2_), 1.53 (s, 8H, 2 × **L2**–*H*_CH2_). ^13^C-NMR (CDCl_3_, 100 MHz, TMS): δ 173.9, 167.5, 156.7, 155.1, 148.1, 137.5, 146.4, 138.2, 137.5, 135.9, 135.8, 135.4, 128.1, 128.0, 124.4, 124.0, 123.5, 123.4, 39.2, 38.8, 34.1, 33.6, 33.5, 33.2, 32.4, 31.2, 27.5, 27.3, 26.6, 26.3, 24.1, 18.6, 17.1. FT-IR (KBr, cm^−1^): 3379 (ν_N–H_, w), 2951 (s), 2866 (m), 1644 (ν_C=N_, m), 1564 (w), 1457 (s), 1362 (m), 1303 (w), 1268 (m), 1195 (m), 1164 (w), 1121 (w), 1088 (m), 1033 (w), 850 (w), 805 (w), 747 (s). Anal. Calcd for C_38_H_47_N_3_: C, 83.62, H, 8.68, N, 7.70; Found: C, 83.89, H, 8.31, N, 8.07.

Ar = 2-(C_8_H_15_)-6-MeC_6_H_3_ (**L3/L3′**). Using a similar procedure and molar ratios to that described for **L1**/**L1′,** but with 2-cyclooctyl-6-methylphenylamine hydrochloride as the amine, **L3**/**L3′** was obtained as a yellow powder (0.21 g, 34.5%) with molar ratio of **L3**/**L3′** = 1/0.28 (detected by ^1^H-NMR). ^1^H-NMR (CDCl_3_, 400 MHz, TMS): δ 8.38 (m, 1H, **L3**–*H*_Py_), 8.24 (d, *J* = 7.6 Hz, 1H, **L3′**–*H*_Py_), 7.68 (d, *J* = 7.3 Hz, 1H, **L3′**–*H*_Py_), 7.63 (d, *J* = 8.0 Hz, 1H, **L3**–*H*_Py_), 7.12 (t, *J* = 5.1 Hz, 2H, 2 × **L3**–*H*_Ar_ and 2 × **L3′**–*H*_Ar_), 7.05–6.96 (m, 2H, 2 × **L3**–*H*_Ar_ and 2 × **L3′**–*H*_Ar_), 6.92 (d, *J* = 7.4 Hz, 2H, 2 × **L3**–*H*_Ar_ and 2 × L3′–*H*_Ar_), 6.35 (s, 1H, **L3′**–*H*_NH_), 4.64 (t, *J* = 6.5 Hz, 1H, **L3′**–*H*_CH=_), 2.99–2.93 (m, 2H, **L3**–*H*_CH2_), 2.78–2.74 (m, 2H, **L3**–*H*_CH2_), 2.38 (s, 3H, **L3′**–*H*_CH3_), 2.34 (t, *J* = 7.4 Hz, 2H, **L3**–*H*_CH2_), 2.29 (s, 2H, **L3**–*H*_CH2_), 2.22 (s, 3H, L3–*H*_CH3_), 2.01 (d, *J* = 6.2 Hz, 6H, 2 × **L3**–*H*_CH3-Ph_), 1.88 (s, 2H, 2 × **L3**–*H*_CH2_), 1.88–1.76 (m, 8H, 2 × **L3**–*H*_CH2_), 1.72–1.59 (m, 8H, 4 × L3–*H*_CH2_), 1.53 (s, 8H, 2 × **L3**–*H*_CH2_). ^13^C-NMR (CDCl_3_, 100 MHz, TMS): δ 173.7, 167.5, 156.6, 155.0, 148.1,147.5, 146.4, 138.2, 137.5, 135.9, 134.4, 134.0, 127.9, 127.8,127.7, 125.3, 125.1, 124.9, 124.6, 123.7, 123.4, 121.7, 118.5, 38.4, 35.5, 34.4, 33.9, 33.3, 32.7, 31.9, 27.2, 26.9, 26.8, 26.6, 26.5, 26.2, 24.0, 18.8, 18.7, 18.4, 17.4. FT-IR (KBr, cm^−1^): 3377 (ν_N–H_, w), 2952 (s), 2865 (m), 1644 (ν_C=N_, m), 1567 (w), 1456 (s), 1363 (m), 1304 (w), 1267 (m), 1198 (m), 1164 (w), 1123 (w), 1090 (m), 1033 (w), 851 (w), 806 (w), 749 (s). Anal. Calcd for C_42_H_55_N_3_: C, 83.81, H, 9.21, N, 6.98; Found: C, 84.12, H, 9.06, N, 6.68%.

Ar = 2-(C_5_H_9_)-6-Me_2_C_6_H_2_ (**L4/L4′**). Using a similar procedure and molar ratios to that described for **L1**/**L1′** but with 2-cyclopentyl-4,6-dimethylphenylamine hydrochloride as the amine, **L4**/**L4′** was obtained as a yellow powder (0.20 g, 36.6%) with molar ratio of **L4**/**L4′** = 1/0.09 (detected by ^1^H-NMR). ^1^H-NMR (CDCl_3_, 400 MHz, TMS): δ 8.39 (t, *J* = 6.9 Hz, 1H, **L4**–*H*_Py_), 8.26 (d, *J* = 8.2 Hz, 1H, **L4′**–*H*_Py_), 7.69 (d, *J* = 7.6 Hz, 1H, **L4′**–*H*_Py_), 7.61 (d, *J* = 8.3 Hz, 1H, **L4**–*H*_Py_), 7.18 (t, *J* = 8.5 Hz, 2H, 2 × **L4**–*H*_Ar_), 7.13 (t, *J* = 5.6 Hz, 2H, 2 × **L4′**–*H*_Ar_), 7.07 (t, *J* = 6.8 Hz, 2H, 2 × **L4**–*H*_Ar_ and 2 × **L4′**–*H*_Ar_), 6.97 (d, J = 7.2 Hz, 2H, 2 × **L4**–*H*_Ar_), 6.82 (d, *J* = 6.7 Hz, 2H, 2 × **L4′**–*H*_Ar_), 6.46 (s, 1H, **L4′**–*H*_NH_), 4.66 (t, *J* = 7.2 Hz, 1H, **L4′**–*H*_CH=_), 3.01–2.94 (m, 2H, **L4**–*H*_CH2_), 2.89–2.78 (m, 2H, **L4**–*H*_CH2_), 2.77 (t, *J* = 6.3 Hz, 2H, **L4′**–*H*_CH2_),2.37 (s, 3H, **L4′**–*H*_CH2_), 2.34 (t, *J* = 6.1 Hz, 2H, **L4**–*H*_CH2_), 2.26 (s, 2H, **L4**–*H*_CH2_), 2.21 (s, 3H, **L4**–*H*_CH3_), 2.01 (d, *J* = 6.2 Hz, 6H, 2 × **L4**–*H*_CH3-Ph_), 1.96 (s, 6H, 2 × **L4**–*H*_CH3-Ph_), 1.88–1.83 (m, 2H, 2 × **L4**–*H*_CH2_), 1.81 (s, 4H, **L4**–*H*_CH2_), 1.74 (t, *J* = 5.7 Hz, 4H, **L4**–*H*_CH2_), 1.61 (d, *J* = 5.3 Hz, 4H, **L4**–*H*_CH2_), 1.54 (s, 4H, **L4**–*H*_CH2_). ^13^C-NMR (CDCl_3_, 100 MHz, TMS): δ 173.4, 167.3, 156.6, 155.1, 147.9, 137.6, 136.2, 134.5, 134.2, 128.1, 127.9, 125.3, 125.3 125.3, 123.8, 124.1, 123.9, 123.7, 123.3, 121.8, 40.6, 40.2, 34.3, 34.1, 33.8, 33.7, 32.4, 31.6, 27.3, 26.3, 26.0, 25.9, 25.8, 24.0, 23.1, 18.5, 17.4, 16.9. FT-IR (KBr, cm^−1^): 3376 (ν_N–H_, w), 2949 (s), 2869 (m), 1646 (ν_C=N_, m), 1565 (w), 1458 (s), 1363 (m), 1307 (w), 1264 (m), 1195 (m), 1164 (w), 1119 (w), 1091 (m), 1033 (w), 849 (w), 804 (w), 747 (s). Anal. Calcd for C_38_H_47_N_3_: C, 83.62, H, 8.68, N, 7.70; Found: C, 83.37, H, 8.92, N, 7.59%.

Ar = 2-(C_6_H_11_)-6-Me_2_C_6_H_2_ (**L5/L5′**). Using a similar procedure and molar ratios to that described for **L1**/**L1′** but with 2-cyclohexyl-4,6-dimethylphenylamine hydrochloride as the amine, **L5**/**L5′** was obtained as a yellow powder (0.22 g, 38.5%) with molar ratio of **L5**/**L5′** = 1/0.12 (detected by ^1^H-NMR). ^1^H-NMR (CDCl_3_, 400 MHz, TMS): δ 8.37 (t, *J* = 8.4 Hz, 1H, **L5**–*H*_Py_), 8.24 (d, *J* = 8.0 Hz, 1H, **L5′**–*H*_Py_), 7.66 (d, *J* = 7.6 Hz, 1H, **L5′**–*H*_Py_), 7.62 (d, *J* = 7.6 Hz, 1H, **L5**–*H*_Py_), 7.14 (t, *J* = 8.1 Hz, 2H, 2 × **L5**–*H*_Ar_ and 2 × **L5′**–*H*_Ar_), 7.02 (m, 2H, 2 × **L5**–*H*_Ar_ and 2 × **L5′**–*H*_Ar_), 6.96 (d, *J* = 7.1 Hz, 2H, 2 × **L5**–*H*_Ar_), 6.93 (s, 2H, 2 × **L5**′–*H*_Ar_), 6.40 (s, 1H, **L5′**–*H*_NH_), 4.65 (t, *J* = 6.9 Hz, 1H, **L5′**–*H*_CH=_), 2.94 (t, *J* = 6.7 Hz, 2H, **L5**–*H*_CH2_), 2.83–2.76 (m, 2H, **L5**–*H*_CH2_), 2.67 (s, 2H, **L5′**–*H*_CH2_), 2.42 (s, 3H, **L5′**–*H*_CH3_), 2.38 (t, *J* = 6.4 Hz, 2H, **L5**–*H*_CH2_), 2.31 (s, 2H, **L5**–*H*_CH2_), 2.26 (s, 3H, **L5**–*H*_CH2_), 2.05 (d, *J* = 5.0 Hz, 6H, 2 × **L5**–*H*_CH3-Ph_), 2.01 (s, 6H, 2 × **L5**–*H*_CH3-Ph_), 1.97 (s, 2H, 2 × **L5**–*H*_CH2_), 1.88–1.82 (m, 4H, **L5**–*H*_CH2_), 1.77–1.65 (m, 8H, 2 × **L5**–*H*_CH2_), 1.54 (s, 8H, 2 × **L5**–*H*_CH2_). ^13^C-NMR (CDCl_3_, 100 MHz, TMS): δ 174.1, 167.8, 156.9, 155.4, 148.2, 137.7, 146.4, 138.3, 137.5, 135.9, 135.7, 135.4, 128.2, 128.0, 124.4, 124.1, 123.5, 123.3, 39.4, 39.0, 34.3, 33.7, 33.4, 33.1, 32.4, 31.2, 27.6, 27.4, 26.5, 26.3, 24.0, 18.6, 17.2. FT-IR (KBr, cm^−1^): 3379 (ν_N–H_, w), 2951 (s), 2864 (m), 1645 (ν_C=N_, m), 1563 (w), 1457 (s), 1364 (m), 1301 (w), 1272 (m), 1194 (m), 1164 (w), 1120 (w), 1089 (m), 1034 (w), 850 (w), 804 (w), 748 (s). Anal. Calcd for C_40_H_51_N_3_: C, 83.72, H, 8.96, N, 7.32; Found: C, 83.45, H, 8.73, N, 7.58%.

Ar = 2-(C_8_H_15_)-6-Me_2_C_6_H_2_ (**L6/L6′**). Using a similar procedure and molar ratios to that described for **L1**/**L1′** but with 2-cyclooctyl-4,6-dimethylphenylamine hydrochloride as the amine, **L6**/**L6′** was obtained as a yellow powder (0.20 g, 32.1%) with crude molar ratio of **L6**/**L6′** = 1/0.21 (detected by ^1^H-NMR). ^1^H-NMR (CDCl_3_, 400 MHz, TMS): δ 8.41 (m, 1H, **L6**–*H*_Py_), 8.27 (d, *J* = 7.6 Hz, 1H, **L6′**–*H*_Py_), 7.70 (d, *J* = 7.3 Hz, 1H, **L6**′–*H*_Py_), 7.66 (d, *J* = 8.0 Hz, 1H, **L6**–*H*_Py_), 7.15 (t, *J* = 6.1 Hz, 2H, 2 × **L6**–*H*_Ar_ and 2 × **L6′**–*H*_Ar_), 7.11–6.99 (m, 2H, 2 × **L6**–*H*_Ar_ and 2 × **L6′**–*H*_Ar_), 6.94 (d, J = 7.7 Hz, 2H, 2 × **L6**–*H*_Ar_ and 2 × **L6′**–*H*_Ar_), 6.38 (s, 1H, **L6′**–*H*_NH_), 4.66 (t, *J* = 6.9 Hz, 1H, **L6′**–*H*_CH=_), 3.07–3.01 (m, 2H, **L6**–*H*_CH2_), 2.81–2.75 (m, 2H, **L6**–*H*_CH2_), 2.42 (s, 3H, **L6′**–*H*_CH3_), 2.35 (t, *J* = 7.4 Hz, 2H, **L6**–*H*_CH2_), 2.33 (s, 2H, **L6**–*H*_CH2_), 2.25 (s, 3H, L6–*H*_CH3_), 2.11 (d, *J* = 6.0 Hz, 6H, 2 × **L6**–*H*_CH3-Ph_), 2.03 (d, *J* = 5.9 Hz, 6H, 2 × **L6**–*H*_CH3-Ph_), 1.90 (s, 2H, 2 × **L6**–*H*_CH2_), 1.88–1.76 (m, 8H, 2 × **L6**–*H*_CH2_), 1.71–1.60 (m, 8H, 4 × **L6**–*H*_CH2_), 1.51 (s, 8H, 2 × **L6**–*H*_CH2_). ^13^C-NMR (CDCl_3_, 100 MHz, TMS): δ 174.6, 168.1, 157.3, 155.5, 148.5,147.9, 146.6, 138.3, 137.8, 136.2, 134.4, 134.1, 128.3, 127.9, 127.7, 125.7, 125.4, 124.9, 124.8, 123.7, 123.6, 121.9, 118.5, 38.4, 36.0, 34.6, 33.9, 33.4, 32.5, 32.0, 27.4, 26.9, 26.7, 26.4, 26.3, 26.0, 24.1, 18.8, 18.6, 18.2, 17.4. FT-IR (KBr, cm^−1^): 3374 (ν_N–H_, w), 2948 (s), 2865 (m), 1644 (ν_C=N_, m), 1565 (w), 1459 (s), 1361 (m), 1303 (w), 1263 (m), 1194 (m), 1165 (w), 1119 (w), 1090 (m), 1036 (w), 851 (w), 805 (w), 749 (s). Anal. Calcd for C_44_H_59_N_3_: C, 83.89, H, 9.44, N, 6.67; Found: C, 83.63, H, 9.26, N, 6.98%.

### 3.3. Synthesis of [2-(1-ArN) C_2_H_3_-9-ArN-5,6,7,8-C_5_H_8_C_5_H_3_N] CoCl_2_ (**Co1**–**Co6**)

Ar = 2-(C_5_H_9_)-6-MeC_6_H_3_ (**Co1**). The ligand **L1**/**L1′** (110.0 mg, 0.23 mmol) and CoCl_2_·6H_2_O (47.3 mg, 0.20 mmol) were dissolved in 5 mL of ethanol. The reaction mixture was stirred at room temperature for overnight after which time diethyl ether (30 mL) was added to precipitate the complex. The precipitate was collected by filtration and washed with diethyl ether (3 × 5 mL), followed by drying under vacuum to give complex **Co1** as a light brown powder (122.0 mg, 94.0%). FT-IR (KBr, cm^−1^): 2947 (s), 2863 (m), 2167 (w), 2138 (w), 2012 (w), 1992 (w), 1608 (ν_C=N_, m), 1571 (s), 1454 (s), 1369 (m), 1309 (w), 1257 (s), 1198 (s), 1113 (m), 1086 (w), 934 (w), 888 (w), 845 (m), 744 (vs). Anal. Calcd for C_36_H_43_Cl_2_CoN_3_: C, 66.77, H, 6.69, N, 6.49; Found: C, 66.42, H, 6.61, N, 6.55%.

Ar = 2-(C_6_H_11_)-6-MeC_6_H_3_ (**Co2**). Using a similar procedure and molar ratios to that described for **Co1** but with **L2**/**L2′** as the ligand, the complex **Co2** was obtained as the light brown powder in 126.0 mg (yield: 93.5%). FT-IR (KBr, cm^−1^): 2923 (s), 2851 (m), 2168 (w), 2119 (w), 2022 (w), 1989 (w), 1606 (ν_C=N_, m), 1570 (s), 1448 (s), 1369 (m), 1314 (w), 1260 (m), 1230 (s), 1194 (s), 1114 (m), 1088 (w), 936 (w), 886 (w), 846 (m), 774 (vs), 736 (w). Anal. Calcd for C_38_H_47_Cl_2_CoN_3_: C, 67.55, H, 7.01, N, 6.22; Found: C, 67.77, H, 6.94, N, 6.34%..

Ar = 2-(C_8_H_15_)-6-MeC_6_H_3_ (**Co3**). Using a similar procedure and molar ratios to that described for **Co1** but with **L3**/**L3′** as the ligand, the complex **Co3** was obtained as the light brown powder in 130.0 mg (yield: 91.2%). FT-IR (KBr, cm^−1^): 2916 (s), 2853 (m), 2167 (w), 2082 (w), 2014 (w), 1989 (w), 1609 (ν_C=N_, m), 1569 (s), 1465 (s), 1368 (m), 1313 (w), 1257 (s), 1193 (s), 1114 (m), 1085 (w), 931 (w), 845 (m), 771 (vs), 735 (w). Anal. Calcd for C_42_H_55_Cl_2_CoN_3_: C, 68.94, H, 7.58, N, 5.74; Found: C, 69.31, H, 7.47, N, 5.89%.

Ar = 2-(C_5_H_9_)-4,6-Me_2_C_6_H_2_ (**Co4**). Using a similar procedure and molar ratios to that described for **Co1** but with **L4**/**L4′** as the ligand, the complex **Co4** was obtained as the light brown powder in 126.0 mg (yield: 93.3%). FT-IR (KBr, cm^−1^): 2916 (s), 2857 (m), 2166 (w), 2078 (w), 2032 (w), 1991 (w), 1609 (ν_C=N_, m), 1569 (s), 1472 (s), 1448 (s), 1368 (m), 1311 (w), 1257 (s), 1205 (s), 1113 (m), 1085 (w), 926 (w), 850 (s), 768 (s). Anal. Calcd for C_38_H_47_Cl_2_CoN_3_: C, 67.55, H, 7.01, N, 6.22; Found: C, 67.82, H, 7.31, N, 6.06%.

Ar = 2-(C_6_H_11_)-4,6-Me_2_C_6_H_2_ (**Co5**). Using a similar procedure and molar ratios to that described for **Co1** but with **L5**/**L5′** as the ligand, the complex **Co5** was obtained as the light brown powder in 135.0 mg (yield: 95.6%). FT-IR (KBr, cm^−1^): 2918 (vs), 2852 (s), 2169 (w), 2111 (w), 2011 (w), 1989 (w), 1611 (ν_C=N_, m), 1569 (s), 1447 (s), 1367 (m), 1313 (w), 1258 (s), 1204 (m), 1116 (w), 1086 (w), 850 (s), 768 (s). Anal. Calcd for C_40_H_51_Cl_2_CoN_3_: C, 68.27, H, 7.31, N, 5.97; Found: C, 67.89, H, 7.47, N, 6.33%.

Ar = 2-(C_8_H_15_)-4,6-Me_2_C_6_H_2_ (**Co6**). Using a similar procedure and molar ratios to that described for **Co1** but with **L6**/**L6′** as the ligand, the complex **Co6** was obtained as the light brown powder in 133.0 mg (yield: 87.4%). FT-IR (KBr, cm^−1^): 2916 (s), 2854 (m), 2166 (w), 2112 (w), 2012 (w), 1990 (w), 1611 (ν_C=N_, m), 1568 (s), 1466 (s), 1441 (s), 1368 (m), 1314 (w), 1257 (m), 1212 (m), 1114 (w), 1084 (w), 926 (w), 852 (s), 768 (s). Anal. Calcd for C_44_H_59_Cl_2_CoN_3_: C, 69.55, H, 7.83, N, 5.53; Found: C, 69.81, H, 7.58, N, 5.85%.

### 3.4. X-ray Crystallographic Studies

Single-crystal X-ray diffraction studies of **Co1** and **Co3** were conducted on a Rigaku Sealed Tube CCD (Saturn 724+) diffractometer with graphite-mono chromated Mo-Kα radiation (λ = 0.71073 Å) at 173(2) K and the cell parameters obtained by global refinement of the positions of all collected reflections. Intensities were corrected for Lorentz and polarization effects and empirical absorption. The structures were solved by direct methods and refined by full-matrix least-squares on F^2^. All non-hydrogen atoms were refined anisotropically and all hydrogen atoms were placed in calculated positions. Structure solution and refinement were performed by using the SHELXT (Sheldrick, 2015) [55]. The disorder displayed by the carbon atoms in **Co3** was processed by the SHELXL-2015 software [56]. Crystal data and processing parameters for **Co2** and **Co3** are summarized in Table 4. X-ray crystallographic data in CIF for the Cambridge Crystallographic Data Centre (CCDC) 1,897,124 (**Co2**) and 1,897,125 (**Co3**) are available free of charge from The Cambridge Crystallographic Data Centre.

### 3.5. General Procedure for Ethylene Polymerization under 5/10 Atm Pressure

A 250 mL stainless steel autoclave (Dalian Sanling Electronic Manufacture, Dalian, China), equipped with an ethylene pressure control system, a mechanical stirrer and a temperature controller, was employed for the reaction. The autoclave was evacuated and refilled with ethylene three times. When the desired reaction temperature was reached, toluene, the co-catalyst (MAO or MMAO), and a toluene solution of the catalytic precursor (the total volume was 100 mL) were injected into the autoclave by using syringes. Then, the ethylene pressure was increased to 5/10 atm, maintained at this level with constant feeding of ethylene. After the reaction was carried out for their quired period, the reactor was cooled with a water bath and the excess ethylene was vented. The reaction solution was quenched with 10% HCl/ethanol. The precipitated polymer was collected through filtration, washed with ethanol and dried under vacuum at 60 °C until constant weight.

## 4. Conclusions

A family of six cobalt (II) chloride complexes, **Co1**–**Co6**, bound by single ring-fused 2-(1-cycloalkylphenylimino)ethyl-9-cycloalkylphenylimino-5,6,7,8-tetrahydrocyclo-heptapyridines, was prepared by the reaction of the corresponding carbocyclic-fused *NNN*-pincer ligands (**L1**/**L1′**–**L6**/**L6′**) and cobalt(II) chloride and fully characterized. On activation with either MMAO or MAO, all these *ortho* cycloalkyl substituted cobalt complexes displayed good activity for ethylene polymerization with the optimum temperature of 50 °C, affording the linear polyethylene. The molecular weight (Mw = 9.78–25.6 kg mol^−1^) is higher than that observed with their analogues **D** bearing alkyl substituent. The ring size of the *ortho* substituent greatly affected their molecular weight and polymerization activity, which was demonstrated by the highest activity achieved by the cyclopentyl substituted ones (**Co1** and **Co4**) and the highest molecular weight by cyclooctyl substituted ones (**Co3** and **Co6**). Notably, polyethylene using MAO as activator displayed high selectivity for vinyl end-groups (–CH=CH_2_), while with MMAO the polyethylene possessed both the saturated and saturated linear structure.

## Figures and Tables

**Table 1:** Selected bond lengths (Å) and angles (°) for **Co2** and **Co3.**

	Co2	Co3		Co2	Co3
Bond Lengths (Å)					
Co(1)–N(1)	2.203(4)	2.193(4)	Co(1)–N(2)	2.063(4)	2.043(4)
Co(1)–N(3)	2.182(3)	2.175(4)	Co(1)–Cl(1)	2.2922(13)	2.3062(15)
Co(1)–Cl(2)	2.2646(12)	2.2478(14)	N(1)–C(2)	1.286(6)	1.296(6)
N(3)–C(11)	1.286(5)	1.296(6)			
Bond Angles (deg)					
N(1)–Co(1)–N(2)	73.80(14)	74.91(16)	N(1)–Co(1)–N(3)	142.70(13)	147.01(15)
N(2)–Co(1)–N(3)	74.65(14)	75.80(16)	N(1)–Co(1)–Cl(2)	97.55(11)	98.74(11)
N(2)–Co(1)–Cl(2)	152.75(11)	151.03(13)	N(3)–Co(1)–Cl(2)	101.45(10)	99.53(11)
N(1)–Co(1)–Cl(1)	100.12(11)	98.37(11)	N(2)–Co(1)–Cl(1)	95.66(11)	89.97(12)
N(3)–Co(1)–Cl(1)	102.30(10)	96.45(12)	Cl(2)–Co(1)–Cl(1)	111.42(5)	119.00(6)
C(11)–N(3)–Co(1)	116.0(3)	113.9(3)	C(2)–N(1)–Co(1)	114.9(3)	114.8(4)

**Table 2:** Ethylene polymerization results using **Co2**/ MAO *^a^_._*

Run	Precat.	Al/Co	T (°C)	t (min)	PE (g)	Act. ^*b*^	*M*_w_ *^c^*	*M*_w_/*M*_n_^*c*^	*T*_m_ (^o^C) ^*d*^
1	**Co2**	1000	30	30	3.66	2.44	22.97	3.7	133.7
2	**Co2**	1000	40	30	3.74	2.49	17.94	3.8	131.4
3	**Co2**	1000	50	30	4.33	2.89	14.21	3.0	131.1
4	**Co2**	1000	60	30	2.70	1.80	9.37	2.0	130.2
5	**Co2**	1000	70	30	1.45	0.96	6.56	2.5	130.1
6	**Co2**	500	50	30	Trace	-	-	-	-
7	**Co2**	1500	50	30	4.37	2.91	9.76	2.4	130.3
8	**Co2**	2000	50	30	4.43	2.95	9.75	2.4	130.7
9	**Co2**	2500	50	30	5.36	3.57	9.39	2.7	130.0
10	**Co2**	3000	50	30	5.10	3.40	10.67	2.6	130.5
11	**Co2**	3500	50	30	1.94	1.29	13.96	2.5	130.6
12	**Co2**	2500	50	5	2.03	8.12	10.65	2.6	130.3
13	**Co2**	2500	50	15	4.70	6.26	10.34	2.5	130.7
14	**Co2**	2500	50	45	6.28	2.78	11.21	2.6	131.5
15	**Co2**	2500	50	60	7.44	2.48	11.26	2.7	130.1
16 *^e^*	**Co2**	2500	50	30	4.28	2.85	13.04	2.7	130.8
17	**Co1**	2500	50	30	6.13	4.09	10.34	2.4	130.4
18	**Co3**	2500	50	30	4.95	3.30	20.20	3.9	132.4
19	**Co4**	2500	50	30	5.25	3.50	13.40	2.4	131.6
20	**Co5**	2500	50	30	4.87	3.25	9.78	2.4	130.2
21	**Co6**	2500	50	30	4.82	3.21	25.96	4.4	132.8

*^a^* Reaction conditions: 3 μmol cobalt complexes, 100 mL toluene, 10 atm C_2_H_4_; *^b^* Values in units of 10^6^ g of PE mol^−1^ (Co) h^−1^; *^c^* Determined using GPC, *M*_w_: kg mol^−1^; *^d^* Determined using DSC; *^e^* 5 atm C_2_H_4_.

**Table 3:** Ethylene polymerization results using **Co2**/MMAO *^a.^*

Run	Precat.	Al/Co	T (°C)	t (min)	PE (g)	Act. *^b^*	*M*_w_ *^c^*	*M*_w_/*M*_n_*^c^*	*T*_m_ (^o^C) *^d^*
1	**Co2**	2500	30	30	3.34	2.23	12.98	2.8	132.5
2	**Co2**	2500	40	30	3.82	2.55	11.42	2.6	130.3
3	**Co2**	2500	50	30	4.43	2.95	8.19	2.5	129.1
4	**Co2**	2500	60	30	3.20	2.13	6.41	2.5	128.1
5	**Co2**	1500	50	30	3.62	2.41	10.22	2.4	130.1
6	**Co2**	2000	50	30	3.75	2.50	9.27	2.7	129.4
7	**Co2**	3000	50	30	2.27	1.51	9.86	2.3	129.9
8	**Co2**	2500	50	5	1.76	7.04	10.49	2.5	130.0
9	**Co2**	2500	50	15	3.98	5.31	8.29	2.5	129.1
10	**Co2**	2500	50	60	6.21	2.07	10.06	2.4	131.2
11 *^e^*	**Co2**	2500	50	30	2.85	1.90	8.90	2.5	129.9
12	**Co1**	2500	50	30	5.01	3.34	10.01	2.4	130.0
13	**Co3**	2500	50	30	3.74	2.49	14.72	2.2	130.9
14	**Co4**	2500	50	30	4.87	3.25	10.79	2.4	129.8
15	**Co5**	2500	50	30	3.98	2.65	9.22	2.6	129.6
16	**Co6**	2500	50	30	3.36	2.24	15.94	4.5	133.3

*^a^* Reaction conditions: 3 μmol cobalt complexes, 100 mL toluene, 10 atm C_2_H_4_; *^b^* Values in units of 10^6^ g of PE mol^−1^ (Co) h^−1^; *^c^* Determined using GPC, *M*_w_: kg mol^−1^; *^d^* Determined using DSC; *^e^* 5 atm C_2_H_4_.

**Table 4:** Crystal data and structure refinement for **Co2** and **Co3**.

	Co2	Co3
CCDC No.	1897124	1897125
Crystal color	Brown	Yellow
Empirical formula	C_38_H_47_Cl2CoN_3_	C_42_H_55_Cl2CoN_3_
Formula weight	675.61	731.72
Temperature (K)	173.15	173.15(2)
Wavelength (Å)	0.71073	0.71073
Crystal system	orthorhombic	tetragonal
Space group	Fdd2	I4_1_/a
a (Å)	33.4157(6)	34.3337(8)
b (Å)	31.4531(6)	34.3337(8)
c (Å)	16.9829(4)	14.5297(7)
α (°)	90	90
β (°)	90	90
γ (°)	90	90
Volume (Å^3^)	17849.5(6)	17127.7(11)
Z	16	16
D_calcd_ (g cm^−3^)	1.006	1.135
μ (mm^−1^)	0.528	0.555
*F*(000)	5712.0	6224.0
Crystal size (mm^3^)	0.288 × 0.193 × 0.102	0.274 × 0.144 × 0.118
*θ* rang (°)	4.56–63.054	3.044–66.158
Limiting indices	−46 ≤ *h* ≤ 48, −43 ≤ *k* ≤ 46, −24 ≤ *l* ≤ 23	−50 ≤ h ≤ 48, −47 ≤ k ≤ 51, −21 ≤ l ≤ 10
No. of rflns collected	70088	49080
No. unique rflns [R(int)]	0.0625(10266)	0.1028(4627)
Completeness to *θ* (%)	93.0 (θ = 25.00)	90.6 (θ = 25.00)
Goodness of fit on *F*^2^	1.040	0.971
Final R indices [I > 2σ(I)]	R_1_ = 0.0625, wR_2_ = 0.1560	R_1_ = 0.1028, wR_2_ = 0.2154
R indices (all data)	R_1_ = 0.0868, wR_2_ = 0.1705	R_1_ = 0.2894, wR_2_ = 0.3034
Largest diff. peak and hole (e Å^−3^)	0.41 and −0.21	0.42 and −0.42

## Abbreviations

PE	Polyethylene
ORTEP	Oak Ridge Thermal Ellipsoid Plot
CIF	Calibration Index File
GPC	Gel Permeation Chromatography
MAO	methylaluminoxane
MMAO	Modified methylaluminoxane
PDI	Polydispersity index
*T*m	Melting temperature
